# Supplementary material for: Making cough count in tuberculosis care
Source: Commun Med (Lond). 2022 Jul 6;2:83. doi: 10.1038/s43856-022-00149-w (PMC9258463; doi:10.1038/s43856-022-00149-w)
Supplement: Supplementary file 2 — Supplementary Information [file 43856_2022_149_MOESM2_ESM.pdf]

Supplementary information

Supplementary Table 1 – Summary table of studies investigating cough in the context of TB care, cough detection, and cough classification

Cough recording tool	Author (Year)	Ref.	Sample size	Setting	Reference standard	Study design and objectives	Algorithm description
**Objective cough monitoring for TB**							
Tape recorder	Loudon (1969)	1	63 TB patients	TB patients hospitalized in Dallas, USA	N/A	Prospective cohort demonstrating that nighttime cough frequency is associated with disease severity	N/A
Leicester Cough Monitor and Visual Analog Scale (VAS)	Turner (2014)	2	108	N/A	Sputum culture, sputum smear microscopy	Retrospective review of medical records of TB patients' cough 24h prior to commencing treatment	N/A
Leicester Cough Questionnaire (LCQ) and Cough and Sputum Assessment Questionnaire (CASA-Q)	Suzuki (2019)	3	85	TB patients hospitalized in Shizuoka, Japan	Sputum culture, sputum smear microscopy	Prospective observational cohort comparing LCQ and CASA-Q score on admission and at discharge	N/A
Leicester Cough Monitor	Turner (2018)	4	44	TB clinics and in-patient facilities in the United Kingdom	Sputum culture, sputum smear microscopy, chest X-ray	Cross-sectional survey of cough frequency in patients with TB and their contacts	N/A

Leicester Cough Monitor	Williams (2020)	5	24	Inpatients admitted to one of three hospitals in Pretoria, South Africa	Liquid sputum culture (BACTEC MGIT 960), sputum smear microscopy, Xpert MTB/RIF, chest X-ray	Prospective cohort over a 24h period to correlate exhaled TB bacillary output with cough frequency	N/A
Cayetano Cough Monitor*	Tracey (2011)	6	62 TB patients	Public national tertiary referral hospital in Lima, Peru	MODS culture, sputum smear microscopy, clinical symptoms	Prospective cohort examining cough pattern and frequency among TB patients prior to treatment and during the first 60 days of treatment.	TB cough detection algorithm using sequential minimal optimization (SMO)<br><br>Sensitivity = 81%<br>Specificity = N/A<br>Overall accuracy = 86.4%
Cayetano Cough Monitor*	Larson (2012)	7	15 TB patients	Tertiary referral hospital in Lima, Peru	N/A	Prospective cohort examining the change in cough frequency during the first 2 weeks of TB therapy	TB cough detection algorithm using sequential minimal optimization (SMO)<br><br>Sensitivity = 75.5%<br>Specificity = 99.6%
Cayetano Cough Monitor*	Proaño (2017)	8	64 TB patients	Two reference tertiary academic hospitals in Lima, Peru	MODS culture, auramine-stained smear	Prospective cohort study evaluating cough patterns among TB patients prior to treatment and during the first 62 days of treatment.	Same algorithm as Larson (2012)[7]
Cayetano Cough Monitor*	Proaño (2018)	9	41	Two tertiary hospitals in Lima, Peru	MODS culture	Prospective cohort study examining the relationship between cough frequency and cavitary lung disease throughout the first 60 days of TB therapy	N/A

Cayetano Cough Monitor*	Lee (2020)	10	71 TB patients	Two tertiary hospitals in Lima, Peru	MODS culture	Prospective cohort examining the change in cough frequency among patients with TB during the first 60 days of TB therapy	Same algorithm as Larson (2012)[7]
**Examples of cough detection AI algorithms**							
Hyfe Cough Tracker smartphone app	Gabaldon-Figueira (2021)	11	57 participants	Community members located within 5km of the University of Navarra, Spain	N/A	Prospective observational study to assess the value of digital acoustic surveillance in predicting respiratory disease incidence (including COVID-19)	Cough detection algorithm using Convolutional Neural Network model (CNN)<br><br>Sensitivity = 96.34%<br>Specificity 96.54%
AI4COVID-19 smartphone app.	Imran (2020)	12	543 coughs	N/A	N/A	Development of COVID-19 cough detection AI model	Sensitivity = 96.01%<br>Specificity = 95.19%
HealthMode Cough smartphone application	Kvapilova (2019)	13	20 people	Online material (including YouTube videos and SoundSnap website)	Manual cough counting	Development of cough detection AI model	Sensitivity = 90% at 99.5% specificity<br>Sensitivity = 75% at 99.9% specificity
**Examples of TB and COVID-19 cough classification AI algorithms**							
TimBre smartphone app.	Pathri (2022)	14	# people<br>TB: 5<br>Non-TB: 469	Tertiary hospital in Bangalore, India	Sputum smear microscopy, Xpert (unspecified), chest X-ray	Development of TB cough classification AI model	TB cough classification algorithm using RUS Boosted Algorithm<br><br>Sensitivity = 80%<br>Specificity = 92%

Tascam DR-44WL hand-held audio recorder and a Rhode M3 microphone	Botha (2018)	15	# people<br>TB: 17<br>Non-TB: 21	Recording done in a "specially designed facility"	Sputum culture	Development of TB cough classification AI model	TB cough classification algorithms using fusion by logistic regression<br><br>Sensitivity = 95% at 72% specificity
ZOOM F8N field recorder and a RØDE M3 condenser microphone	Pahar (2021)	16	# people<br>TB: 16<br>Control: 35	Primary health care clinic in Cape Town, South Africa	"Bacteriological TB diagnosis"	Development of TB cough classification AI models	TB cough classification algorithms using logistic regression (LR), support vector machines (SMV), k-nearest neighbor (KNN), multilayer perceptron's (ML), and convolutional neural networks (CNN)<br><br>LR performed best:<br>Sensitivity = 93%<br>Specificity = 95%
AI4COVID-19 smartphone app.	Imran (2020)	12	# coughs<br>COVID: 70<br>Non-COVID: 473	N/A	N/A	Development of COVID-19 cough classification AI model	COVID-19 cough classification algorithms using Deep Transfer Learning-based Multi-Class (DTL-MC), Classical Machin Learning-based Multi Class (CML-MC), Deep Transfer Learning-based Binary Class (DTL-BC) classifiers<br><br>DTL-MC<br>Sensitivity = 89.14%<br>Specificity = 96.67%<br><br>CML-MC<br>Sensitivity = 91.71%

							Specificity = 95.27%<br><br>DTL-BC<br>Sensitivity = 94.57%<br>Specificity = 91.14%
MIT Open Voice Initiative website (opensigma.mit.edu)	Laguarta (2020)	17	# people<br>COVID: 2660<br>Non-COVID: 2660	Global cough collection through online platform:	"Official test", doctor assessment, or personal assessment	Development of COVID-19 cough classification AI model	COVID-19 cough classification algorithm using a Convolutional Neural Network (CNN) model<br><br>Sensitivity = 98.5%<br>Specificity = 94.2%
Android phones and web apps by University of Cambridge (covid-19-sounds.org./en/)	Coppock (2021)	18	# people<br>COVID: 62<br>Non-COVID: 293	Crowdsourced participants	Self-reporting	Development of COVID-19 cough classification AI model	COVID-19 cough classification algorithm using a Deep Neural Network (DNN) model<br><br>AUC = 0.846

* Cayetano Cough Monitor is a Marantz PMD 620 handheld recorder with an Audio-Technica AT899 sub-mini microphone attached at the patient's lapel
TB = tuberculosis; AI = artificial intelligence; Ctl = control; MODS = microscopic-observation drug-susceptibility; N/A = not available

Supplementary references

1. Loudon, R. G. & Spohn, S. K. Cough frequency and infectivity in patients with pulmonary tuberculosis. *Am. Rev. Respir. Dis.* **99**, 109–111 (1969).
2. Turner, R., Repossi, A., Matos, S., Birring, S. & Bothamley, G. S79 Cough Prevalence And Frequency In Pulmonary Tuberculosis. *Thorax* **69**, A43–A44 (2014).
3. Suzuki, T. *et al.* Improved cough- and sputum-related quality of life after initiation of treatment in pulmonary tuberculosis. *Respir. Investig.* **57**, 252–259 (2019).
4. Turner, R. D. *et al.* Daily cough frequency in tuberculosis and association with household infection. *Int. J. Tuberc. Lung Dis.* **22**, 863–870 (2018).
5. Williams, C. M. *et al.* Exhaled Mycobacterium tuberculosis output and detection of subclinical disease by face-mask sampling:

prospective observational studies. *Lancet. Infect. Dis.* **20**, 607–617 (2020).
6. Tracey, B. H. *et al.* Cough detection algorithm for monitoring patient recovery from pulmonary tuberculosis. *Annu Int Conf IEEE Eng Med Biol Soc* **2011**, 6017–6020 (2011).
7. Larson, S. *et al.* Validation of an Automated Cough Detection Algorithm for Tracking Recovery of Pulmonary Tuberculosis Patients. *PLoS One* **7**, e46229 (2012).
8. Proaño, A. *et al.* Dynamics of cough frequency in adults undergoing treatment for pulmonary tuberculosis. *Clin. Infect. Dis.* **64**, 1174–1181 (2017).
9. Proaño, A. *et al.* Cough Frequency During Treatment Associated With Baseline Cavitary Volume and Proximity to the Airway in Pulmonary TB. *Chest* **153**, 1358–1367 (2018).
10. Lee, G. O. *et al.* Cough dynamics in adults receiving tuberculosis treatment. *PLoS One* **15**, e0231167 (2020).
11. Carlos Gabaldon-Figueira, J. *et al.* Digital acoustic surveillance for early detection of respiratory disease outbreaks in Spain: a protocol for an observational study. *BMJ Open* **11**, e051278 (2021).
12. Imran, A. *et al.* AI4COVID-19: AI enabled preliminary diagnosis for COVID-19 from cough samples via an app. *Informatics Med. Unlocked* **20**, 100378 (2020).
13. Kvapilova, L. *et al.* Continuous Sound Collection Using Smartphones and Machine Learning to Measure Cough. *Digit. Biomarkers* **3**, 166–175 (2019).
14. Pathri, R., Jha, S., Tandon, S. & GangaShetty, S. Acoustic Epidemiology of Pulmonary Tuberculosis (TB) & Covid19 leveraging AI/ML. *medRxiv* 2022.02.05.22269707 (2022) doi:10.1101/2022.02.05.22269707.
15. Botha, G. *et al.* Detection of tuberculosis by automatic cough sound analysis. *Physiol. Meas.* **39**, 045005 (2018).
16. Pahar, M. *et al.* Automatic Cough Classification for Tuberculosis Screening in a Real-World Environment. *Physiol. Meas.* **42**, 105014 (2021).
17. Laguarta, J., Hueto, F. & Subirana, B. COVID-19 Artificial Intelligence Diagnosis Using Only Cough Recordings. *IEEE Open J. Eng. Med. Biol.* **1**, 275–281 (2020).
18. Coppock, H. *et al.* End-to-end convolutional neural network enables COVID-19 detection from breath and cough audio: a pilot study. *BMJ Innov.* **7**, 356–362 (2021).
